# Secondary bacterial infection caused by ST16 NDM-1 and OXA-48-producing colistin and carbapenem-resistant *Klebsiella pneumoniae* treated with tigecycline in a pregnant woman with COVID-19

**DOI:** 10.1186/s40545-023-00547-y

**Published:** 2023-03-03

**Authors:** Samaneh Pourajam, Azam Zafarbakhsh, Majid Hosseinzadeh, Mohammad Shirzadi, Mansour Siavash, Hamid Solgi

**Affiliations:** 1 Department of Internal Medicine, School of Medicine, Isfahan University of Medical Sciences, Isfahan, Iran; 2 Department of Obstetrics and Gynecology, School of Medicine, Isfahan University of Medical Sciences, Isfahan, Iran; 3 Department of Genetics and Molecular Biology, School of Medicine, Isfahan University of Medical Sciences, Isfahan, Iran; 4 Isfahan Endocrine and Metabolism Research Center, Isfahan University of Medical Sciences, Isfahan, Iran; 5 Division of Clinical Microbiology, Department of Laboratory Medicine, Amin Hospital, Isfahan University of Medical Sciences, Isfahan, Iran

**Keywords:** Superinfection, Carbapenem and colistin-resistant *Klebsiella pneumoniae* and Tigecycline

## Abstract

During the COVID-19 pandemic, the rapid emergence of carbapenem and colistin-resistant *Klebsiella pneumoniae* has resulted in an alarming situation worldwide. We aimed to describe secondary infections and antimicrobial use, in a pregnant woman admitted to hospital with COVID-19. A 28-year-old pregnant woman was admitted to the hospital due to COVID-19. According to the clinical conditions, the patient was transferred to the ICU on the second day. She was empirically treated with ampicillin and clindamycin. Mechanical ventilation through an endotracheal tube was started on the 10th day. During her hospitalization in the ICU, she was infected with *ESBL*-producing *K. pneumonia*, *Enterobacter* spp and carbapenemase-producing colistin-resistant *Klebsiella pneumoniae* isolates. Finally, the patient was treated with tigecycline monotherapy that was associated with *ventilator-associated pneumonia* clearance. Bacterial co-infection is relatively infrequent in hospitalized patients with COVID-19. Treatment of infections caused by carbapenemase-producing colistin-resistant *K. pneumoniae* isolates is challenging, with limited antimicrobials available in Iran. In order to prevent the spread of extensively drug-resistant bacteria, infection control programs must be implemented more seriously.

## Introduction

Antimicrobial resistance during the COVID-19 pandemic is highly associated with the presence of *Klebsiella pneumoniae*, mainly carbapenemase-producing isolate [1–3]. Our previous report revealed that the prevalence of carbapenem-resistant *K. pneumoniae* (CRKP) had increased during the first wave of the pandemic in Iran [4]. Many studies showed that the percentage of CRKP infections increased during the COVID-19 pandemic in accordance with our previous study [1, 2]. It is interesting to note that during the fifth wave of COVID-19 associated with the delta variant, we faced the emergence of colistin and carbapenem-resistant *K. pneumoniae* (Col-CRKP) in our hospital. Here, we reported a 28-week pregnant woman of COVID-19 associated acute hypoxemic respiratory failure who was initially treated with corticosteroids, remdesivir and tocilizumab, and was successfully liberated from mechanical ventilation and then successfully treated with tigecycline.

## Case presentation

**Fig. 1:**
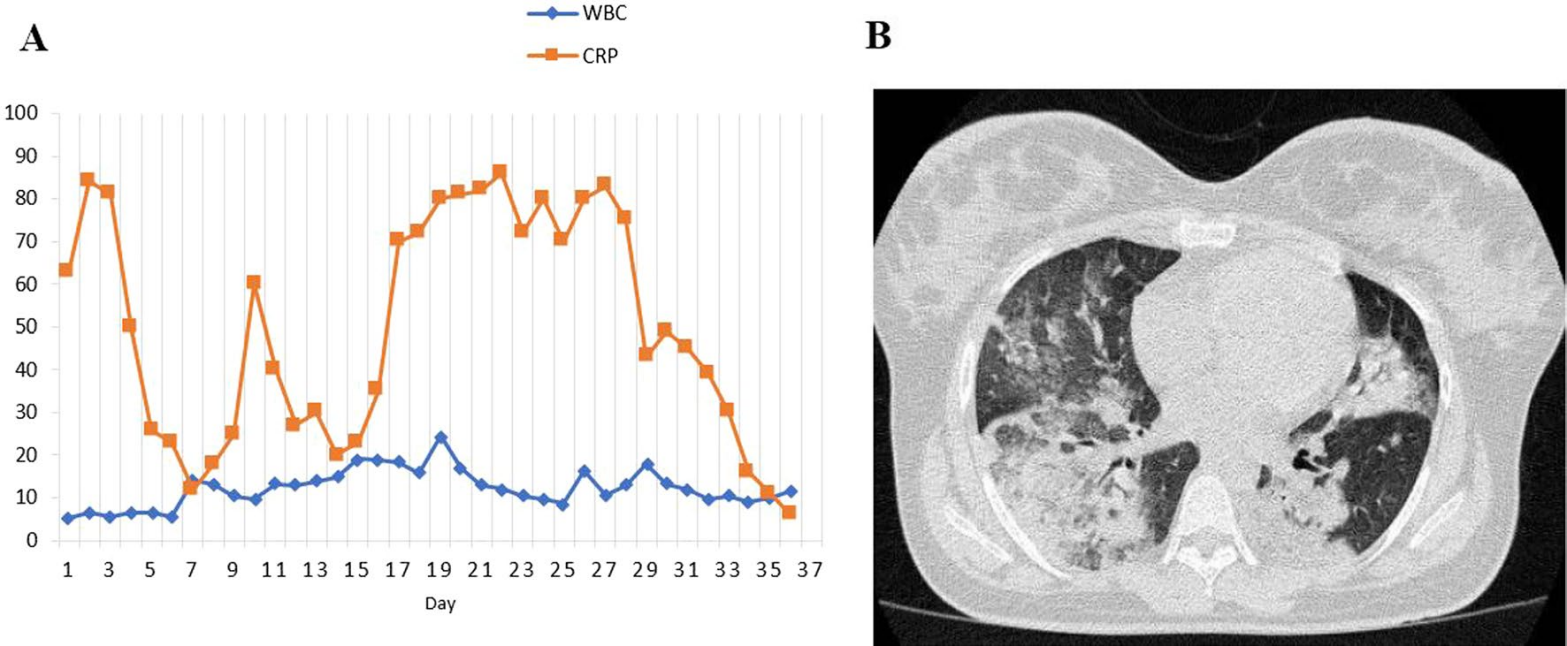
**A** The leukocyte (10^3^/uL) and CRP (mg/dl) trend chart of the patient. **B** Chest computed tomography scan showing bilateral ground-glass opacity

On August 3, 2021, a 28-year-old woman, 28 weeks pregnant with a BMI (body mass index) of 30.5 kg/m^2^, visited the emergency ward for productive cough, weakness, fever, and shortness of breath for 3 days during an outbreak of domestic COVID-19. The patient was a housewife, and she does not have any past medical history. At the beginning of admission, her saturation of peripheral oxygen was 83%, and with the reservoir bag mask had been 94%. Her laboratory data showed a white blood cell (WBC) count of 5200/μL, lymphocyte 20.5%, C-reactive protein (CRP) 63 mg/dl, D-dimer 1912 mg/dl, and Cr 0.6 mg/dl. Figure 1A shows the WBC and CRP trend chart of the patient.

**Fig. 2:**
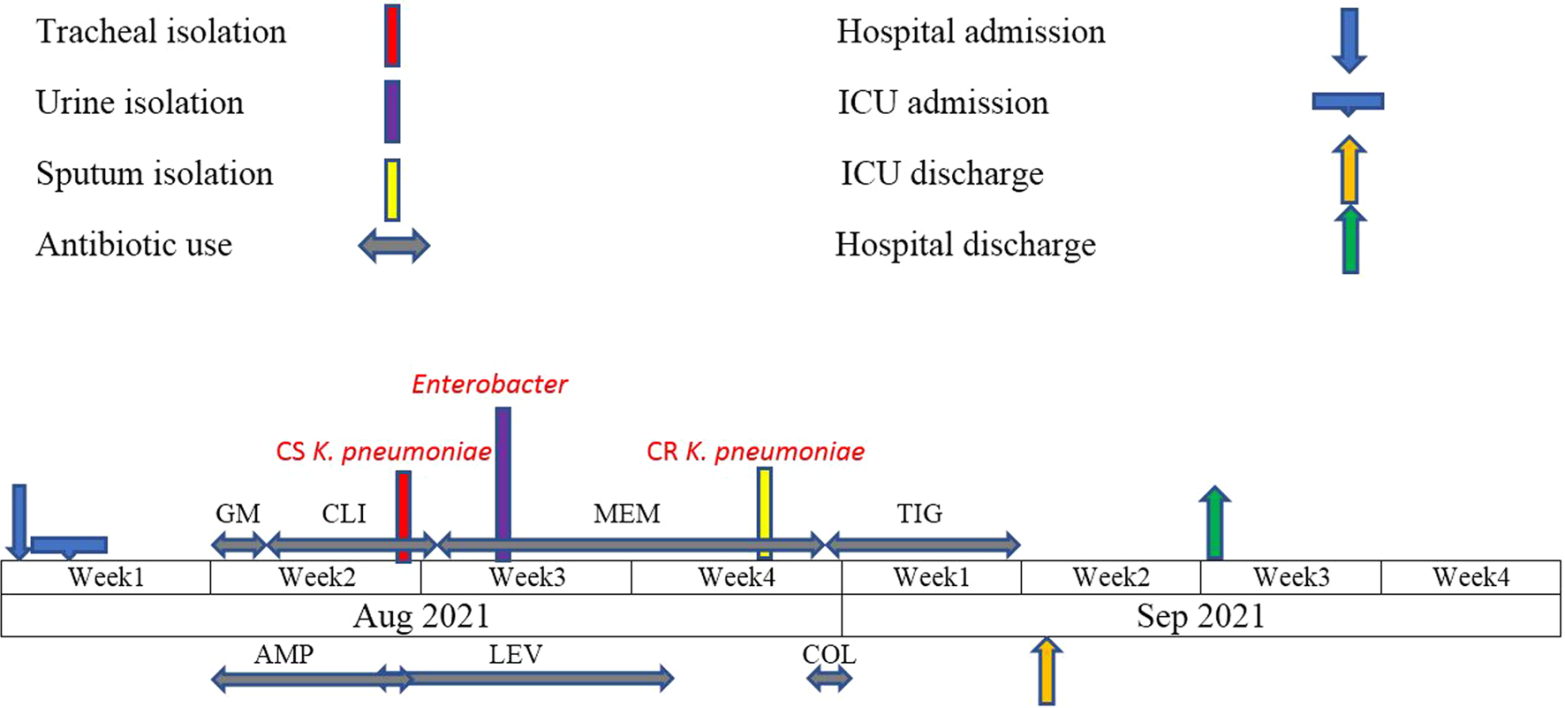
Treatment process from the admission to the discharge of the patient. *GM* gentamicin, *AMP* ampicillin, *CLI* clindamycin, *LEV* levofloxacin, *MEM* meropenem, *COL* colistin, *TIG* tigecycline

The SARS-CoV-2 reverse transcriptase-polymerase chain reaction was positive. The chest MDCT (multi-detector computed tomography) showed more than 50% bilateral ground-glass opacities (Fig. 1B). After being admitted, dexamethasone and remdesivir therapy were started on the first day. On the second day, the patient was admitted to the intensive care unit due to her special condition. Since, O2 saturation with the reservoir bag mask was 85% and with NIV use was 90%, so, one dose of tocilizumab 600 mg was given alongside. On the 7th day of admission, ampicillin and gentamicin were prescribed. The antibiotic treatment process of the patient is shown in Fig. 2.

On day seven, following a prolonged nonreactive non-stress test (NST) and placental abruption cesarean section (C/S) was performed in the negative-pressure operating room under spinal anesthesia, and a 2400-g female infant was delivered with an Apgar score of 6/10 and 8/10 in the first and fifth minute after delivery. Two days after the cesarean section, the patient had a generalized tonic–clonic seizure. Serum creatinine was raised to 2.4 mg/dl, lactate dehydrogenase (LDH) reached 4064 U/L, and prothrombin time was also elevated. Peripheral blood smear showed progressive thrombocytopenia (64,000 × 103/μL) and *schistocytes*. Plasmapheresis with FFP replacement was performed and continued until thrombotic thrombocytopenic purpura was ruled out. The following anuria and decreased GFR hemodialysis was initiated in the next day.

On the 10th day of hospitalization, she was obtunded and oxygen saturation dropped to 80% on NIV. Mechanical ventilation was initiated through an endotracheal tube. The next day she became febrile (peak at 40 ℃) but we did not find an incisional infection or discharge. There were abundant purulent thick lung secretions. So, the first blood, urine, and tracheal aspirate samples were taken for culture. Empiric therapy with meropenem was initiated to cover suspected nosocomial infection, and then ampicillin and clindamycin were discontinued. The endotracheal culture yielded *ESBL*-producing *K. pneumonia* isolate which was almost resistant to all the antibiotics except for colistin, meropenem, and amikacin, and her urine sample was positive for an *Enterobacter* spp. strain sensitive to colistin, meropenem, amikacin, and nitrofurantoin, therefore meropenem continued.

According to the clinical improvement of the patient's thrombocytopenia and the report of normal ADAMTS13 activity, plasmapheresis was stopped. The patient had tachycardia and moderate right atrial and right ventricle enlargement and pulmonary arterial pressure was increased to 37 mmHg in echocardiography. Heparin with therapeutic dose was infused for high probable pulmonary thromboembolic (PTE). She initially improved and was liberated from mechanical ventilation, while her Glasgow Coma Scale (GCS) was 8 T (with NIV o2 saturation around 90%), but on day 25, again, an increase in the volume of sputum and infection indicators were observed (fever, 39.5 ℃; PCT, 2.4 ng/mL and CRP, 63 mg/dl), which was suspected to be a new pneumonia infection. Therefore, a new sputum culture was performed, revealing a CRKP isolate (minimum inhibitory concentrations [MICs] ≥ 32) that was also resistant to amikacin (MIC ≥ 16 μg/ml), and colistin (MIC 16 μg/ml) and sensitive only to tigecycline (MIC 1 μg/ml). Our genomic study showed that the isolated strain carried carbapenemase (*bla*_NDM-1_ and *bla*_OXA-48_) and ESBL (*bla*_CTX-M_, *bla*_SHV,_ and *bla*_TEM_) genes and belonged to *ST16*.

Since the tigecycline is not in our hospital pharmacopeia list, we had one day delay for tigecycline infusion, so meropenem plus colistin was prescribed till tigecycline was prepared. After the preparation of tigecycline, the antibiotics were changed to intravenous tigecycline on the 28th day. Since her clinical condition had improved, the antibiotic regimen was stopped after 10 days of tigecycline administration. Meanwhile, two consecutive sputum cultures from hospital day 32 yielded negative results. On the 31st day of the hospital, she was extubated and on the 36th day, the patient was transferred to the general ward for functional recovery. Scan perfusion reported a high probability of acute PTE subsequently warfarin was prescribed. Her oxygen requirement decreased and after 42 days she was discharged with nasal oxygen, warfarin, and tapering prednisolone.

## Discussion

Infections caused by CRKP isolates often have few treatment options and are associated with high mortality rates in hospitalized patients with COVID-19 [5]. Superinfections caused by CRKP strains have been increasingly reported during the hospital stays of patients with severe COVID-19 [4–7]. In our previous study conducted during the first wave of the pandemic, we found that the prevalence of pulmonary superinfection caused by CRKP isolates increased duration of ICU hospitalization [4].

This is the first case report describing the microbiological improvement of lung infection caused by ST16 Col-CRKP with tigecycline treatment in Iran during the fifth wave of the COVID-19 pandemic. In our country, the last line of treatment for infections caused by CRKP isolates is colistin and tigecycline, because new β-lactam/β-lactamase combinations, such as ceftazidime–avibactam, meropenem–vaborbactam, and imipenem–relebactam are not available in the Iranian pharmacopoeia list. The EUCAST recommends tigecycline susceptibility breakpoints in Enterobacteriaceae of susceptible MIC ≤ 1 mg/L and resistant MIC > 2 mg/L(r). Therefore, according to the MIC of meropenem (MIC ≥ 32 μg/ml), colistin (MIC 16 μg/ml) and tigecycline (MIC 1 μg/ml), and also because of the limited therapeutic options, an infectious disease specialist performed monotherapy treatment with tigecycline for 10 days and then the patient was discharged after 42 days.

In the literature, cases treated with tigecycline monotherapy in lung infections due to Col-CRKP are limited. A study by Lin et al*.* from Taiwan showed that tigecycline monotherapy was not associated with a higher mortality among patients with monotherapy [8], which is similar to this report. However, increasing evidence has suggested that combination therapy against CRKP is more effective than monotherapy and leads to better outcomes. Therefore, antibiotic monotherapy for infections caused by Col-CRKP isolates may contribute to the failure of certain antibiotic treatments [5, 9, 10]. The spread of Col-CRKP in ICU wards is concerning and highlights the need for infection control measures in healthcare settings.

## Conclusion

In conclusion, we described the characteristics and outcome of a patient with COVID-19 who developed a superinfection by Col-CRKP, highlighting that the spread and dissemination of this pathogen occurring in the ICU wards represents a relevant challenge for clinicians in Iran due to the limited treatment options available.

## Data Availability

All data generated or analyzed during this study are included in this article.

## Abbreviations

CRKP: Carbapenem-resistant *K. pneumoniae*
Col-CRKP: Carbapenem-resistant *K. pneumoniae*
BMI: Body mass index
WBC: White blood cell
CRP: C-reactive protein
MDCT: Multi-detector computed tomography
NST: Non-stress test
C/S: Cesarean section
LDH: Lactate dehydrogenase
PTE: Pulmonary thromboembolic
GCS: Glasgow Coma Scale
MIC: Minimum inhibitory concentrations
